# Unraveling Dravet Syndrome: Exploring the complex effects of sodium channel mutations on neuronal networks

**DOI:** 10.1177/00368504231225076

**Published:** 2024-02-19

**Authors:** Nina Doorn

**Affiliations:** 1 Department of Clinical Neurophysiology, 3230 University of Twente, Enschede, The Netherlands

**Keywords:** Dravet Syndrome, SCN1A, epilepsy, computational modeling, stem cells

## Abstract

Dravet Syndrome (DS) is a severe developmental epileptic encephalopathy with frequent intractable seizures accompanied by cognitive impairment, often caused by pathogenic variants in *SCN1A* encoding sodium channel Na_V_1.1. Recent research utilizing *in vitro* patient-derived neuronal networks and accompanying *in silico* models uncovered that not just sodium—but also potassium—and synaptic currents were impaired in DS networks. Here, we explore the implications of these findings for three questions that remain elusive in DS: How do sodium channel impairments result in epilepsy? How can identical variants lead to varying phenotypes? What mechanisms underlie the developmental delay in DS patients? We speculate that impaired potassium currents might be a secondary effect to Na_V_1.1 mutations and could result in hyperexcitable neurons and epileptic networks. Moreover, we reason that homeostatic plasticity is actively engaged in DS networks, possibly affecting the phenotype and impairing learning and development when driven to extremes.

## Introduction

Dravet Syndrome (DS) is one of the most prevalent and severe developmental epileptic encephalopathies, characterized by frequent seizures and a high risk of sudden unexpected death in epilepsy.^1^ The seizures are often unresponsive to anti-epileptic drugs,^2^ and accompanied by cognitive impairment, developmental delay, and motor dysfunction.^3,4^ In 80% of the cases, DS is caused by a *de novo* variant in *SCN1A,* which encodes the voltage-gated sodium channel Na_V_1.1 α-subunit.^3^ However, patients with the same variant show substantial variability in phenotype and treatment response. How impaired sodium currents can lead to epilepsy is still an enigma as epilepsy is considered a “hyperexcitability problem.” Observations in animal models of DS suggest reduced sodium currents in inhibitory neurons, but not excitatory neurons, are involved in the generation of epileptic seizures through disinhibition.^5–9^ However, recent advances in human-induced pluripotent stem cell (hiPSC) derived neurons have complicated this observation by implicating the contribution of excitatory neurons to the DS phenotype.^10,11^ Besides incomplete understanding of epileptogenesis in DS, the origin of the phenotype variability also remains elusive. DS patients with the same pathogenic variant, even within the same family, can show different clinical phenotypes, ranging from seizure-free to severe forms of DS.^2,12^ Studies investigating correlations between *SCN1A* variants and phenotype severity have not found a reliable correspondence.^13–15^ Another unanswered question concerns mechanisms underlying the developmental delay in language, motor function, learning, and social skills that heavily affect many DS patients. To our knowledge, no linkage between the type of *SCN1A* variant and cognitive outcome has been found,^4,16^ nor between environmental factors and cognitive outcome.^17^ In our recent paper, we approach DS in a novel way using both a human neuronal network model and an accompanying biophysical computational model that allows to obtain mechanistic insights.^1^ The insights obtained with this approach might further our understanding regarding the three questions mentioned above: how can sodium channel impairments result in epilepsy, how can identical variants result in varying phenotypes, and what mechanisms underlie the developmental delay in DS patients? In this commentary, we speculate about the possible implications our previous work has for answering these questions.

## Reduced potassium currents and synaptic strengths in DS networks

In our recent paper, we used hiPSCs derived from a DS patient with a heterozygous missense variant in the pore domain of *SCN1A,* to develop electrically mature neuronal networks. These networks allowed us to investigate the effect of the *SCN1A* variant on human network activity, which has some advantages compared to animal models or single-neuron models of epilepsy.^18^ We studied the spontaneous network activity with multi-electrode arrays (MEAs). The activity of DS-derived neuronal networks significantly differed from the activity of networks derived from healthy individuals. This difference was characterized by less and longer synchronous network bursts (NBs) with more random spiking activity in between. To complement our *in vitro* model, we developed a biophysical computational model that can be used to obtain pathophysiological insights into the neuronal networks by simulating the activity on MEA. We extensively validated our computational model and compared its performance to cultures under different circumstances to show how the model can correctly identify the effect of specific cellular changes on the network activity. We used this model to investigate which cellular mechanisms might underly the DS-network phenotype we observed on MEAs. Our hypothesis was that a particular change in the dynamics of the sodium channel should be able to replicate this phenotype, since the patients’ variant is limited to *SCN1A*. To this end, we systematically varied all parameters affecting the sodium channel dynamics. In particular, we modified the conductivity, voltage sensitivity, rates of activation and inactivation, time constants and the contribution of the persistent sodium current and evaluated the effects on simulated network activity. Surprisingly, none of those changes or combinations thereof replicated the experimentally observed DS-network phenotype. Specifically, while the increased NB rate (NBR) could be simulated by altering the sodium channel dynamics, the increased NB duration (NBD) and the lower percentage of spikes in NBs (PSIBs) could not. This suggests that, if the model is correct, sodium channel alterations alone are insufficient to explain the phenotype of DS-derived neuronal networks on MEA. We therefore explored additional changes in neuronal properties. First, we investigated the increased NBD. The termination of the NBs in our model mainly results from the slow afterhyperpolarizing (sAHP) potassium current. Therefore, longer NBs could indicate lower sAHP currents. The lower PSIB means there is more activity that is not correlated between the electrodes, hinting at network desynchronization. Therefore, lower PSIB might be due to less or weaker connections between the neurons. Indeed, lowering the amount of sAHP currents and the synaptic strengths resulted in simulations similar to the experimental DS-network phenotype. We then aimed to confirm these model predictions in the *in vitro* neuronal networks. In the simulations, a lower sAHP resulted in hyperexcitable neurons. Similarly, we found a reduced rheobase and higher firing rates in the *in vitro* neurons. We also measured spontaneous excitatory postsynaptic currents and found their amplitudes to be significantly reduced in DS-neurons, indicative of lower synaptic strengths. Those results suggest that besides sodium channel alterations, potassium currents and synaptic strengths are reduced in our DS-patient-derived neuronal networks. What might that imply for the questions asked above about the epileptogenesis, phenotype variability, and developmental delay?

## Hyperexcitable neurons through impaired potassium currents

The first challenging question is how reduced functioning of a major brain sodium channel can result in epilepsy, i.e. a hyperexcitable network. While our *in vitro* DS-neurons also showed reduced sodium current densities, neuronal excitability was increased. Many *in vitro SCN1A* models show these reduced sodium current densities, yet paradoxically increased neuron excitability, which is incompletely understood.^19–22^ In our computational model, the increased excitability resulted from reduced sAHP potassium currents. These smaller potassium currents could result from reduced sodium currents, since sodium-dependent potassium currents underly part of the sAHP.^23–25^ Other large contributors to the sAHP are the slow calcium-activated potassium currents. These currents could be decreased by reduced calcium influx during action potentials (APs) because of the lower AP amplitude we observed *in vitro*. A similar reduction of AP amplitude was observed in a *Scn2a* knockout mouse model, where Spratt et al.^26^ explained neocortical pyramidal cell hyperexcitability by a reduction in hyperpolarizing potassium currents. Using an *in silico* model, they showed that sodium channel alterations led to differences in depolarization dynamics, which in turn prevented potassium channels from properly repolarizing neurons between subsequent APs. This eventually led to increased excitability because neurons could reach the threshold for subsequent APs more rapidly. Moreover, van Hugte et al.^27^ showed that there was an increased afterhyperpolarization time and reduced AHP potential change in SCN1A^+/−^ -deficient human neurons, indicative of reduced AHP currents. Additionally, they showed a significant decline in AP decay dynamics, indicative of altered potassium channel dynamics. More evidence for the involvement of potassium channels came from Jorge et al.,^28^ who showed that Kv8.2 potassium channels function as a genetic modifier in *Scn2a* mutant mice leading to pyramidal cell hyperexcitability, and Reid et al.,^29^ who rescued pyramidal cell hyperexcitability with potassium channel opener Retigabine. Moreover, two studies^9,30^ showed reduced expression of the Na_V_ β1.1 subunit protein, which modulates multiple potassium currents,^31,32^ in *Scn1a* deficient mice. Taken together, reduced potassium currents as a secondary effect to reduced sodium currents might explain excitatory neuronal hyperexcitability in DS or similar sodium channel-related disorders.

## Phenotype variability through differential homeostasis

The second challenging question is what candidate mechanism are involved in the high phenotype variability in DS. It has been speculated that factors besides Na_V_1.1 modify the phenotype, but little such factors have been identified.^33^ In our recent paper, we argue that the reduced synaptic strengths in DS neuronal networks might result from homeostatic synaptic downscaling. Synaptic downscaling is a form of homeostatic plasticity, a group of mechanisms to promote stability in neuronal firing rates.^34^ Homeostatic plasticity has been shown to be present in this type of *in vitro* neuronal networks.^35,36^ Moreover, reduced synaptic conductances and expressional changes of synaptic transmission proteins have been reported in DS mice, indicative of homeostatic synaptic mechanisms.^37,38^ We hypothesize that in our DS-networks, neuronal network activity is persistently elevated due to neuronal hyperexcitability and that as a result, synapses are downscaled in order to maintain stable firing rates. The idea that homeostatic plasticity is largely active in DS networks might explain the large variability in the phenotype of patients with the same variant. The amount of homeostatic plasticity might vary per patient based on genetic background, or other factors. Xie et al.^19^ used neurons derived from pairs of CRISPR/Cas9-edited iPSC lines to distinguish between the effect of *SCN1A* variants and genetic background. They found many neuronal and network changes to be dependent on genetic background, including changes in PSC amplitudes, which they attributed to homeostatic regulations. Thus, we speculate that homeostatic plasticity might be a factor that can modify the phenotype and cause differences between patients.

## Impaired learning through downregulated synapses

The third question is how pathogenic sodium channel variants can lead to problems in cognitive development and intellectual disability later in life. There are many sodium channelopathies associated with neurodevelopmental disorders, but we do not fully understand the biological mechanisms connecting the two.^39,40^ Learning and memory are associated to synaptic processes such as Hebbian learning, and most neurodevelopmental disorders are related to variants in genes encoding synaptic machinery.^41^ Therefore, it is peculiar that in the case of DS, sodium channel alterations that are not related to the synapse lead to similar learning and development problems. Swann et al.^42^ argue that homeostatic mechanisms are actively engaged in the epileptic brain, trying to re-establish normal neuronal network activity. In some forms of intractable epilepsies, like DS, seizures are so intense and frequent that these mechanisms cannot restore normal activity levels. Nevertheless, homeostatic mechanisms remain active and could become maladaptive, meaning the mechanisms are driven to such extremes that they induce undesirable effects. A number of studies have reported decreases in dendrite length and branching complexity in mouse models where seizures were artificially induced.^43–45^ These mice with induced seizures were also learning impaired later in life. Swann et al. argue that in an attempt to re-establish normal network excitability, homeostatic mechanisms limit branching complexity and synapse formation, leading to a reduced amount of functional glutamatergic synapses. These synapses undergo Hebbian plasticity which is an essential mechanism of memory formation. Thus, homeostatic mechanisms may limit the ability to learn by eliminating some of the substrates for learning. In our recent paper, we also reported less complex neuron morphology in our *in vitro* DS networks. We thus speculate that learning impairments in DS patients are not a direct consequence of the *SCN1A* variant, but a consequence of homeostasis driven to extremes.

Although our speculations are based on one cell line from a single patient, similar neuronal network activity on MEA was observed in cell lines from multiple patients with the same and other variants in *SCN1A*, and in a CRISPR/Cas9-edited SCN1A^+/−^ -deficient cell line.^27^ All these networks showed longer or repeated NBs, indicative of reduced adaptive potassium currents, and a significantly lower NBR and PSIB, indicative of a less synchronous network and thus reduced connectivity. Therefore, some of the mechanisms identified in our DS-networks might be applicable to similar networks. Nevertheless, we should keep in mind that these types of *in vitro* neuronal networks constitute only one type of excitatory cell, generated through a time-optimized protocol that skips many developmental steps, and that these networks lack the structure and organization of a human brain. In reality, the effect of sodium channel mutations is much more complex due to the variable expression of the nine mammalian sodium channel α subunit genes throughout the brain and its different neuron types, and throughout development. For example, Goff et al.^46^ showed that selective *Scn1a* deletion in only one type of interneurons resulted in abnormal behavioral development in mice, and Almog et al.^47^ showed how excitatory and inhibitory neurons were differentially affected throughout development. Thus, it is important to keep in mind that the potential outcome of an *SCN1A* mutation for brain function might be affected by where the sodium channel is expressed, what is wrong with the sodium channel, how that alters firing patterns and network connectivity, and when those defects become functionally apparent and change throughout development.^48^

## Conclusion

Despite these limitations, we provide some food for thought concerning possible mechanisms of action in DS or related disorders. We speculate that impaired potassium currents, as a secondary effect to the pathogenic *SCN1A* variant, might cause hyperexcitable neurons and circuits. Furthermore, we suggest that homeostatic synaptic plasticity in response to this hyperexcitability might underly the phenotype variability and developmental delay in DS. Moreover, we show how computational models are an imperative tool to accompany experimental work, in order to uncover complex effects and interactions that might otherwise be overlooked.
